# Which aspects of the everyday behavior of older dogs correlate with performance on a visuo-spatial memory test and the canine cognitive dysfunction rating scale (CCDR)?

**DOI:** 10.3389/fnagi.2026.1678032

**Published:** 2026-02-03

**Authors:** E. García, P. Darder, J. Argüelles, A. Salas-Mani, C. Torre, E. Apper, J. Bowen, J. Fatjó

**Affiliations:** 1 Department of Psychiatry (Affinity Foundation Chair for Animals and Health), Autonomous University of Barcelona, Bellaterra, Spain; 2 Ethogroup Ltd., Barcelona, Spain; 3 Ethoclinic Ltd., Valencia, Spain; 4 School of Veterinary Medicine, Cardenal Herrera University, Valencia, Spain; 5 Affinity Petcare S.A, Barcelona, Spain; 6 The Royal Veterinary College, University of London, London, United Kingdom; 7 Ethometrix Ltd., Hove, United Kingdom

**Keywords:** aging, behavioral assessment, canine cognitive dysfunction, everyday behavior, family dog, functionality, visuo-spatial memory tasks

## Abstract

The behavioral aspects of aging in the domestic dog have primarily been investigated through owner-reported scales that measure specific behavioral signs of aging, and laboratory-based memory and cognitive tests. We need to know more about how aging affects everyday behavior and functionality in owned dogs. This study tested a methodology for identifying patterns in everyday physical and behavioral function associated with behavioral tests and scales. Fifty-seven family dogs aged 8+ years were included. Owners completed the Canine-Cognitive-Dysfunction-Rating-Scale (CCDR), and each dog underwent a visuo-spatial memory test (VSMT) and a physical examination. Owners also completed a 112-item checklist of everyday behaviors and activities that a normally functioning dog should be able to perform. Feature selection was performed using a series of orthogonal projections to latent structures (OPLS) models that sequentially excluded checklist items with low variable importance to projection until R^2^Y and Q^2^ were optimized. All OPLS models were strong and significant (R^2^Y up to 0.579, *p* < 0.0001), and resulted in three short (8–11 item) subscales from the checklist whose scores correlated with their relevant test counterpart: CCDR score (r = −0.63, *p* < 0.0001), VSMT score (r = 0.658, *p* < 0.0001), and physical health score (r = −0.649, *p* < 0.0001). The content of the subscales provided valuable insights into the everyday behavioral correlates of the tests. For example, a pattern of items describing mood, motivation, mobility, vision, memory and trainability was associated with better VSMT performance, but better performance in the CCDR scale was associated with items relating to mobility, exercise tolerance, vision, and hearing. This indicates that in older dogs a substantial proportion of the variability of the results of tests like the VSMT and CCDR, can be accounted for by physical and sensory health issues, and that patterns of everyday behavior are correlated with these tests. We also created an OPLS model for age, as a reference point for comparison. Our results indicate that using multivariate statistics to perform feature selection can identify systematic relationships between everyday behavior and tests and clinical scales, to provide valuable insights into the real-world effects of brain aging.

## 1 Introduction

Companion dogs live in a demanding environment in which they are expected to be obedient, emotionally stable, adaptable, complementary to the lives of their human families and beneficial to society at large. The overall ability to function as a companion dog therefore involves many elements, including sociability with people and dogs, trainability, being able to spend time alone, and accepting different kinds of handling. A dog that functions well would perform well across a wide range of situations that are commonly experienced by companion dogs as part of their everyday lives. Behavioral tests and rating scales are regularly used in scientific studies and, more recently, as part of the evaluation of clinical cases. These can include behavioral tests of memory (Piotti et al., 2022), arousal and self-control (Bray et al., 2015), and rating scales that measure frustration (McPeake et al., 2019), impulsiveness (Wright et al., 2011), anxiety (Mills et al., 2020a), dimensions of personality or temperament (Ley et al., 2009. Salonen et al., 2021) and help to evaluate conditions like canine cognitive dysfunction (CCD) (Ciurli et al., 2024). There are also questionnaires that characterize the human animal bond (Howell et al., 2017) and people’s attachment to pets (Lund et al., 2025).

Although tests and scales provide valuable information, we know relatively little about how their results relate to dogs’ everyday functioning from the family’s perspective. It would be interesting, and useful, to understand the relationship between performance in behavioral tests or rating scales and a dog’s ability to function in everyday life. In essence, to have a functional phenotype that characterizes the everyday performance of dogs with particular personality traits such as anxiousness, frustration or impulsiveness, as well as dogs living with other behaviorally relevant conditions such as chronic pain, sensory decline, cognitive decline, or specific problems such as separation-related issues, and even dogs that people find difficult or easy to form bonds with.

The purpose of this study was therefore to test a methodology for using a wide-ranging general checklist of behavior and function combined with multivariate statistics to identify functional phenotypic patterns of behavior associated with a previously studied and validated behavioral test and rating scales. This approach builds on our earlier work using multivariate methods to investigate the effects of maternal care on puppy behavior (Guardini et al., 2016; Guardini et al., 2017).

Older dogs are ideal for testing a methodology based on functional phenotypes. From around eight years of age onwards, family dogs show substantial inter-individual variability in mobility, sensory function, motivation, emotional stability, and cognitive performance. This heterogeneity provides a rich behavioral landscape in which multivariate methods can detect latent patterns that would be difficult to identify in a more homogeneous population. In addition, validated behavioral and cognitive tools already exist for this life stage, providing objective reference points against which to interpret functional patterns derived from everyday behavior. Importantly, aging affects multiple domains simultaneously, making older dogs an ideal natural model for examining how day-to-day functioning maps onto performance in behavioral tests and rating scales, regardless of whether the underlying processes are cognitive, sensory, emotional, or physical (Schütt et al., 2015; Szabó et al., 2016).

The study of the aging process in both humans and companion animals has garnered increasing attention. In human research, this focus is largely due to the global rise of aging in populations, which poses significant challenges for healthcare systems worldwide (Chen et al., 2025). In dogs, the motivation in research is driven by a desire to enhance companion animal welfare and explore their potential as models for studying human aging (Ruple et al., 2022).

Although the lifespan of both humans and dogs has increased, this does not necessarily translate into a prolonged period of good health. Aging often brings about declines in behavioral and cognitive functions, contributing to neurodegenerative diseases that substantially impact the quality of life (Turcsán and Kubinyi, 2024; Bickenbach et al., 2023). Research conducted on laboratory animals indicates that age-related cognitive impairments in dogs are associated with neuropathological changes, which bear similarities to those observed in humans, including the build-up of *β*-amyloid protein in the brain (Dewey et al., 2019).

Some dogs develop Canine Cognitive Dysfunction (CCD), a condition thought to be analogous to Alzheimer’s Disease (AD) in humans, making dogs a valuable model for investigating human aging (Mihevc and Majdič, 2019; Dewey et al., 2019). CCD is a neurobehavioral condition that primarily affects dogs over eight years old, leading to progressive neurodegenerative changes in the cerebral cortex and hippocampus (Chapagain et al., 2018). These changes result in cognitive decline, which manifests as distinct alterations in behavior and routine activities (Ciurli et al., 2023). Dogs affected by CCD show neuropathological signs such as amyloid-beta (Aβ) deposits, brain atrophy, neuron loss, and reduced neurogenesis, which are similar to those found in humans with AD (Ciurli et al., 2023). Studies involving laboratory Beagles have revealed that visuo-spatial function may start to decline as early as six to seven years of age (Studzinski et al., 2006). Recent advances in identifying biomarkers for CCD (Kim et al., 2024) could help in validating behavioral scales and questionnaires, offering more objective means of studying aging in dogs. The standardization and optimization of behavioral testing protocols to develop efficient and accessible tools for practicing veterinarians remains a practical challenge (Kubinyi and Iotchev, 2020).

Recently, research has shifted focus from laboratory to pet dogs, recognizing that dogs and humans share common environments, such as exposure to pollutants and infection risks, and often have similar lifestyles (Szabó et al., 2016). Pet dogs are therefore considered to be a more suitable model than laboratory dogs for studying the physiological and psychological changes that accompany aging, and to test various preventive approaches that may hinder cognitive decline (Chapagain et al., 2018).

This shift highlights the potential for pet dogs to serve as more ethical and technically appropriate models for human diseases and the aging process, compared to traditional laboratory animals. Pet dogs are increasingly viewed as valuable alternatives to rodents and non-human primates in aging research. Studying the environmental factors associated with aging in dogs may yield insights that are relevant to human aging (Hua et al., 2016).

Clinically, the symptoms of CCD have been summarized by the DISHAAL acronym: disorientation, altered social interactions, sleep–wake cycle disturbances, house soiling, activity level changes, anxiety, and difficulty learning (Haake et al., 2024). A major issue in managing canine aging is that owners often do not report early signs of cognitive dysfunction to veterinarians (Landsberg, 2024). This is particularly true when these signs are subtle or when behavioral changes represent an improvement in previously problematic behaviors. For example, a reduction in activity and destructiveness in a previously over-active and destructive dog. Even when owners do notice these signs, they may mistakenly attribute them to normal aging, failing to recognize their significance for the dog’s health and wellbeing (Landsberg, 2024). Additionally, veterinary teams may not always have the necessary training to recognize and interpret these behavioral changes (Landsberg, 2024), which highlights the need for suitable tools to measure aging process to help pet owners and veterinary professionals.

In veterinary practice, efforts to establish standardized assessment tools for canine cognitive dysfunction (CCD) have been ongoing for years. Several questionnaires—including the canine cognitive dysfunction rating scale (CCDR) (Salvin et al., 2010), the Canine Dementia Scale (CADES) (Madari et al., 2015) and the Canine Cognitive Assessment Scale (CCAS) (Le Brech et al., 2022) —as well as additional screening instruments, have been developed to support the detection, diagnosis, and follow-up of the disorder. Subsequent studies evaluating these tools (Schütt et al., 2015; Ozawa et al., 2019; Fefer et al., 2022; Kim et al., 2024; Haake et al., 2024; Smith and Mills, 2025) indicate that the CCDR demonstrates relatively high sensitivity, and it appears particularly effective in identifying more severe cases of CCD (Haake et al., 2024). The CCDR, developed by Salvin et al., quantifies the severity of CCD and has been validated, demonstrating a positive predictive value of 77.8%, a negative predictive value of 99.3%, and high test–retest reliability (Salvin et al., 2011). Epidemiological studies estimate CCD prevalence at 14.2% in dogs over eight years of age, contrasting sharply with the substantially lower veterinary diagnosis rate of 1.9% (Salvin et al., 2010). Overall, reported prevalence ranges from 14.2 to 22.5% in senior dogs, with a pronounced increase associated with advancing age (Chapagain et al., 2018).

In addition to scales, behavioral tests have also been developed for cognitive assessment in dogs. An early example is the food-searching task described by González and colleagues (2013), caried out in a clinical setting. Since then, several test batteries have been created and refined, including the Mini Mental Test (Kubinyi and Iotchev, 2020), the Modified Vienna Canine Cognitive Battery (MVCCB) published by Chapagain et al. (2020), and the cognitive test series developed by the Dog Aging Project, with contributions from Watowich et al. (2020) and Hargrave et al. (2024). Although these assessments were not designed for conventional laboratory conditions, some were developed within research facilities but specifically for use with pet dogs, such as the Working Memory Task (Fefer et al., 2022) and the Impossible Task (Khan et al., 2023). Others were intended for home-based evaluation (e.g., Hoel et al., 2021) or for clinical application, such as the VSMT (Visuo-Spatial Memory Test) by Piotti and colleagues or the Food Search Test by González and colleagues (Piotti et al., 2022; González-Martínez et al., 2013). An important practical consideration across all cognitive assessments is the time required for owners to complete the tasks, which can range from 2–5 h to less than 15 min depending on the protocol. Several studies have also combined cognitive testing with biological measurements and questionnaires, such as gaze assessments and pNfL concentrations (e.g., Fefer et al., 2022).

For this study, we selected the canine cognitive dysfunction rating scale (CCDR) as a tool that is highly specific for owner-reported signs of cognitive dysfunction, and the Visuo-Spatial Memory Test (VSMT), a short behavioral test that can be administered without the dog undergoing any pretraining and is therefore well suited to clinical environments. The findings from tests and scales that are used to detect and evaluate CCD can easily be confounded by non-cognitive factors such as pain, mobility and sensory issues, so being able to evaluate these factors adds context and interpretability of results. In addition, different tests assess CCD from different theoretical perspectives. The VSMT should measure a very specific aspect of memory, but the items of the CCDR predominantly focus on impaired recognition, disorientation, and confusion. Our approach could add context to show what dogs that perform poorly in these evaluations might look like in daily life. This has the potential to assist in the development of novel tests and scales in the future.

For the purpose of this study, we refer to functioning as the animal’s ability to adapt and perform within its environment, particularly as a companion animal (Fatjó and Bowen, 2020a). This involves evaluating the dog’s quality of life and its role in the human-animal bond, including both the positive and negative impacts on the owner’s lifestyle. Functioning assessments should not only focus on identifying problems but also on capturing successes. For instance, a dog may have an active social life, engage in activities like agility training, or provide emotional support to its owner. These positive behaviors can reflect the dog’s motivations and suggest ways to improve its quality of life and relationship with its owner (Fatjó and Bowen, 2020a).

A novel, comprehensive *ad hoc* inventory of day-to-day situations was used as a source of information about functioning. We refer to this as the Global Functioning Checklist (GFC). This was used as a substrate for the multivariate statistics, to give us insights into the real-world implications of these tests, and to enable us to develop subscales of GFC that were specific to VSMT and CCDR.

The aim of the present study was to answer two main questions:

- Could multivariate statistics be used to identify phenotypic patterns of everyday behaviors that were systematically associated with validated tests of brain aging?
- What additional information could the short subscales generated using this approach give us about those other tests, including what kinds of dogs would perform well or badly in them, and what practical applications might they have?

## 2 Materials and methods

### 2.1 Ethics

The study was granted ethical approval by the Ethics Committee of Cardenal Herrera University in Valencia, Spain (CEEA 20/007). All procedures were conducted in compliance with national and EU legislation, as well as institutional guidelines, and adhered to the International Society for Applied Ethology guidelines for the use of animals in research.

Dog owners were informed that the aim of the study was to enhance understanding of the aging process in pet dogs, and their dogs were given a free health evaluation, including a physical examination and routine blood biochemistry and hematology, as compensation for participating in the study. Had the physical examination and blood tests revealed a serious health issue, the affected dog would have been excluded from the study.

The principal investigator at each study site thoroughly explained all aspects of the procedures involved in the study to the owners, who subsequently signed a written consent form indicating their voluntary participation. Owners were informed that they could withdraw their dogs from the study at any point during the testing process.

The principal investigator at each location was responsible for halting the procedure if the dog exhibited any signs of distress and/or pain beyond discomfort. The study received financial support from Affinity Petcare SA.

### 2.2 Subjects

Recruitment of dogs was carried out by staff at three veterinary clinics located in three different areas over a four-month period. The clinics were approximately 100 km apart. Clinic staff screened their databases to identify dogs from recent previous visits that were aged eight years or older. The inclusion criteria for the study required that dogs be 8 years of age or older, free from acute conditions, diseases, or significant sensory or mobility impairments that would prevent them from performing the tests. Additionally, for welfare reasons, the dogs had to be unafraid of people.

During case selection, prior to contacting the owners, we excluded dogs that did not meet the inclusion criteria. However, we wanted the population to be representative of typical pet dogs of that age group, so we expected them to have minor health issues.

Owners of eligible dogs were contacted, informed about the study and recruited to participate until a total of 20 dogs had been enrolled at each clinic. The only benefit to owners for participation was that their dog had a detailed physical examination, blood tests and a consultation about their dog’s health, at no cost to themselves. No dogs had to be excluded as a result of the findings of those examinations.

In total, 60 dogs participated in the study; however, data from only 57 were included in the analyses due to missing questionnaire data for three dogs (owners had not completed a substantial part of one or more questionnaires). This was unexpected and was only picked up at the time of the analysis.

Once the owners were recruited, the clinic provided the researchers with the necessary information to contact the owners directly to schedule appointments for the study procedures at the clinic.

### 2.3 Procedure

All assessments were carried out by three veterinarians working in three different clinics that belong to the same collaborative group. These clinicians already routinely engage in inter-clinic benchmarking, with reviews of their clinical criteria and procedures, through regular meetings and joint case discussions.

The study was conducted in a designated room within each clinic, specially prepared for this purpose. The room was free of large furniture and any materials or objects that could pose a risk to the dogs or people. This space was never used for clinical procedures. Throughout the entire process, the dogs remained off-leash and were accompanied by their owners.

The protocol consisted of:

1. Written consent for blood extraction (vet staff) and blood extraction (vet staff)
2. Written consent (researcher)
3. Global Functioning Checklist (GFC)
4. CCDR Scale
5. Visuo-Spatial Memory Test (VSMT)
6. Health Problem Score (HPS)
7. Routine blood biochemistry and haematology

All documentation, consent forms and questionnaires were supplied to the owner in printed form, along with a pen, to allow the owner to complete the form during the appointment.

#### 2.3.1 Written consents for blood extraction and the use of excess material for research purposes

The veterinary staff explained the procedure for the blood extraction to the owners and obtained their signed consent in an examination room at the clinic. Following this, the staff accompanied the owners to the study room.

#### 2.3.2 Written consent

The principal investigator at the clinic introduced themselves to the owner, explained all the procedures, and invited the owner to sign the written consent. This process took place in a quiet room.

#### 2.3.3 Global functioning checklist (GFC)

The Global Functioning Checklist (GFC) was designed to provide a snapshot of a dog’s current level of adaptation to its environment and its ability to function as a domestic pet. The 112 items in the checklist capture a dog’s performance in a wide range of common everyday situations. Each item is a short statement such as “*My dog always knows where to look to find his toys*” or “*My dog can easily jump into a car*,” and the owner answers yes or no. The statements detail a dog’s responses to a range of everyday situations including interactions with people and other dogs, reactions to noise, unfamiliar environments, separation from the owner, travel, visitors, obedience, control around wildlife, handling. Taken together, they reveal information about a dog’s core capacities in memory, cognition, sensory perception, motivation, and behavioral–emotional regulation.

Additionally, the questionnaire includes statements regarding the dog’s vision, hearing, mobility, and physical fitness. The checklist includes items that vary in cognitive complexity, capturing information about both simple daily activities, such as walking 1 kilometer, and more complex tasks, such as remaining calm near wildlife (see Supplementary File 1 for the complete questionnaire).

Plain language is used, and the situations would be familiar to any dog owner, making it straightforward for owners to complete without requiring interpretation. The checklist was designed to be used with dogs of any age, not specifically older dogs.

The list of statements was developed through several stages: first, an initial list was proposed by JB, JF, and EG; second, the list was circulated among all the authors, who were asked to add additional statements or comment on the existing ones; third, a face-to-face panel discussion was held to finalize the list of statements. Statements were all positively framed, as this approach has been found to increase engagement and completion rates with longer questionnaires. The GFC took approximately 5–10 min to complete. Dichotomous responses (yes/no) were used to make the otherwise lengthy questionnaire easier and quicker to answer. This response format has also been used successfully in other well-established psychometric tools, such as the Eysenck Personality Questionnaire (EPQ), where it supports rapid completion and minimizes ambiguity in respondent interpretation (Colledani et al., 2018).

The psychometric properties of the GFC were not investigated as it was intended purely as a substrate for the multivariate statistics.

To score the GFC, 1 point is awarded for each affirmative response provided by the owner, yielding a score range of 0 to 112 points.

#### 2.3.4 Canine cognitive dysfunction scale (CCDR)

The researcher provided the owner with the scale printed, along with a pen, to allow the owner to complete the form.

The validated Canine Cognitive Dysfunction Rating (CCDR) scale consists of 13 questions that assess both the frequency and intensity of various behaviors (Salvin). The total score ranges from 16 to 80 points, categorizing dogs into three groups: no signs of CCD, at risk of developing CCD, and displaying signs of CCD. The CCDR scale is widely used in research on canine aging. A recent study compared this scale with other CCD scales (CADES and CCAS) within the same population and concluded that the CCDR is particularly effective in detecting severe cases of CCD (Haake et al., 2024). Other studies have also used this tool to assess cognitive impairment or to compare it with other measures of aging (Kim et al., 2024; Smith and Mills, 2025; Simon et al., 2025).

#### 2.3.5 Visuo-spatial memory test (VSMT)

Piotti and colleagues developed an easy-to-apply visuo-spatial short-term memory test that does not require prior training (Piotti et al., 2017). This test was subsequently validated for reliability (Piotti et al., 2022). The test is conducted in an empty room, where five identical containers are placed on the floor, equidistant from each other, and arranged in a semi-circle at regular intervals. All containers are positioned at an equal distance from a predetermined location, 2 meters away. At the start of each trial, the owner stands at this predetermined location, holding the dog on a leash. The dog observes an experimenter baiting one of the five identical containers and is then walked out of the room. After a 30-s distraction task (such as petting or playing with the dog), the dog is brought back into the room, released from the leash, and allowed to approach the containers. The experimenter records the dog’s first choice and notes the severity of any errors made. The test is repeated once for each container, with the order of baited locations counterbalanced across participants. This test is not designed to be conducted at home, but it is a suitable tool for measuring visuo-spatial memory in pet dogs, in one single session, as it does not require prior training. Scoring was the same as in the original study by Piotti: Dogs scored 0 for a failure (not solving the task at all), 1 for several errors (2–4), 2 for a single error and 3 for success with no errors. Each dog performed five trials and a mean score was calculated.

Before the start of data collection, the first author conducted structured training sessions with the other two examiners to present the memory test protocol step-by-step, clarify any doubts, and agree on standardized procedures for test administration and scoring. This process was intended to promote methodological consistency and reduce inter-examiner variability across centers. Further, the use of orthogonal signal correction, as described in the statistical methods below, removes unwanted variation from data that is not correlated the property of interest, and thereby reduced the influence of potential confounds on multivariate models.

#### 2.3.6 Health problem score (HPS)

As part of the compensation to the owners for participating in the study each dog was given a comprehensive, detailed physical examination. The examination, which was standardized to a checklist that was agreed between the clinicians prior to the study, included a routine physical assessment, evaluation of the stage of periodontal disease, and the use of the Colorado State University Canine Acute Pain Scale (CSU-CAPS). All body parts were examined, including feet, ears, eyes and skin. The heart and lung fields were auscultated, and the dog was observed whilst walking in the clinic. The veterinarian recorded any abnormalities in a checklist. Although the examination was primarily intended to be of help to the client, it was also used as a secondary check to detect any dogs with significant underlying health issues. Urinalysis was not performed because sample collection is difficult for owners and cystocentesis is quite an invasive procedure for a routine health screen. However, we decided that it was worth converting the data into a score that we could use in multivariate modelling, as a comparison to the VSMT and CCDR models. One point was awarded for each condition detected during the physical examination, resulting in a score ranging from 0 to 58 (the full list of items included in the score is in the Supplementary Files). If the examination revealed any issues, one point was added for each condition identified. The total score indicated the number of checklist items that were not within normal limit to obtain the Health Problem Score. The purpose of this score was not to diagnose specific diseases, but rather to obtain a comprehensive overview of the animal’s health, considering factors that might affect the dog’s behavior or mood.

#### 2.3.7 Routine blood biochemistry and hematology

The results of the routine blood biochemistry and hematology were part of the complimentary health assessment of the dog and were not included in the analysis for this study.

### 2.4 Statistics

Basic demographic data (age, weight and breed) of the population was collected. The mean and standard deviation for age, weight, and the various scores for each test (CFS, VSMT, CCDR, HPS) were calculated (Graphpad Prism 10).

Orthogonal projections to latent structures (OPLS) (Trygg and Wold, 2002), was chosen as the method for multivariate modelling because its results are easy to interpret and the integral orthogonal signal correction filter effectively rejects systematic variation in the data that is unrelated to the dependent (Y) variable. This ability to reject uncorrelated variation was important because the measures we were investigating were related both to each other and to an underlying biological process of aging. In addition, the variable importance to projection values generated by OPLS models enabled us to perform feature selection based on both systematic contribution of variables to the model and the association between the independent (X) and dependent variables (Y).

For the multivariate analysis, a series of consecutive models was constructed using OPLS, with variables that had a value for variable importance to projection (VIP) lower than 1 being excluded after each round. This is a recognized method of feature selection in multivariate models (Farrés et al., 2015; Mehmood et al., 2020) and has the effect of selecting variables based on their systematic contribution to the model. An integrated orthogonal signal correction filter was included in the PLS modelling because this rejects systematic variance in the X-block that is not correlated to Y and thereby produces models with a single predictive component. In other words, orthogonal signal correction identifies and separates the part of the data (X) that is unrelated to the target variable (Y) and then subtracts this unwanted, orthogonal variation from the original data prior to multivariate modelling. In each case the GFC data was used as the X-block and the relevant test variable (CCDR, mean VSMT score and health score) were used as Y. All models were created using the correlation matrix. The purpose of OPLS is to identify whether there is any systematic relationship between the X and Y variables, and feature reduction using this method would identify those items in GFC that not only were associated with the Y variable but also were systematically related with each other.

The process of consecutive modelling and feature elimination was stopped when model quality was optimized, with values for R^2^Y and Q^2^ being maximal. R^2^Y provides a measure of the proportion of variance in Y that can be explained by a linear combination of X variables. It indicates the model’s “goodness of fit” from 0 (no fit) to 1 (perfect fit). Q^2^ is an indication of the vulnerability of a model to the removal of data or observations. Ideally, Q^2^ should be a minimum of half of the value of R^2^Y. Low values of Q^2^ indicate that a model has been unduly influenced by a small number of observations or variables, without which the model would collapse. The significance of the models was calculated using the analysis of variance of the cross-validated residuals (CV-ANOVA) (Eriksson et al., 2008). Software used was Sartorius Simca.

The same procedure was used to identify GFC items that were associated with the difference between cases classified as affected or unaffected by CCDR using the published score cutoffs. In this case, orthogonal projections to latent structures discriminant analysis (OPLS-DA) was used.

The distribution of scores was tested with the D’Agostino and Pearson test, and suitable parametric or non-parametric contrasts and correlation tests were chosen (Graphpad Prism 10). Correlations between each of the scores was tested using a Spearman Rank correlation test, as data was not normally distributed. All inter-score correlations were calculated and a Bonferroni corrected threshold for *p* was calculated. This approach was chosen, rather than presenting selected correlations, to avoid type 1 errors.

In addition, a comparison was made between affected and unaffected dogs, classified according to the CCDR cutoff points, for each of the main test scores and GFC model scores using the Mann–Whitney test. As with the inter-score correlations, all inter-group contrasts were calculated and a Bonferroni corrected threshold for *p* was calculated. Again, this approach was chosen, rather than presenting selected contrasts, to avoid type 1 errors.

## 3 Results

### 3.1 Demographics

**Figure 1:**
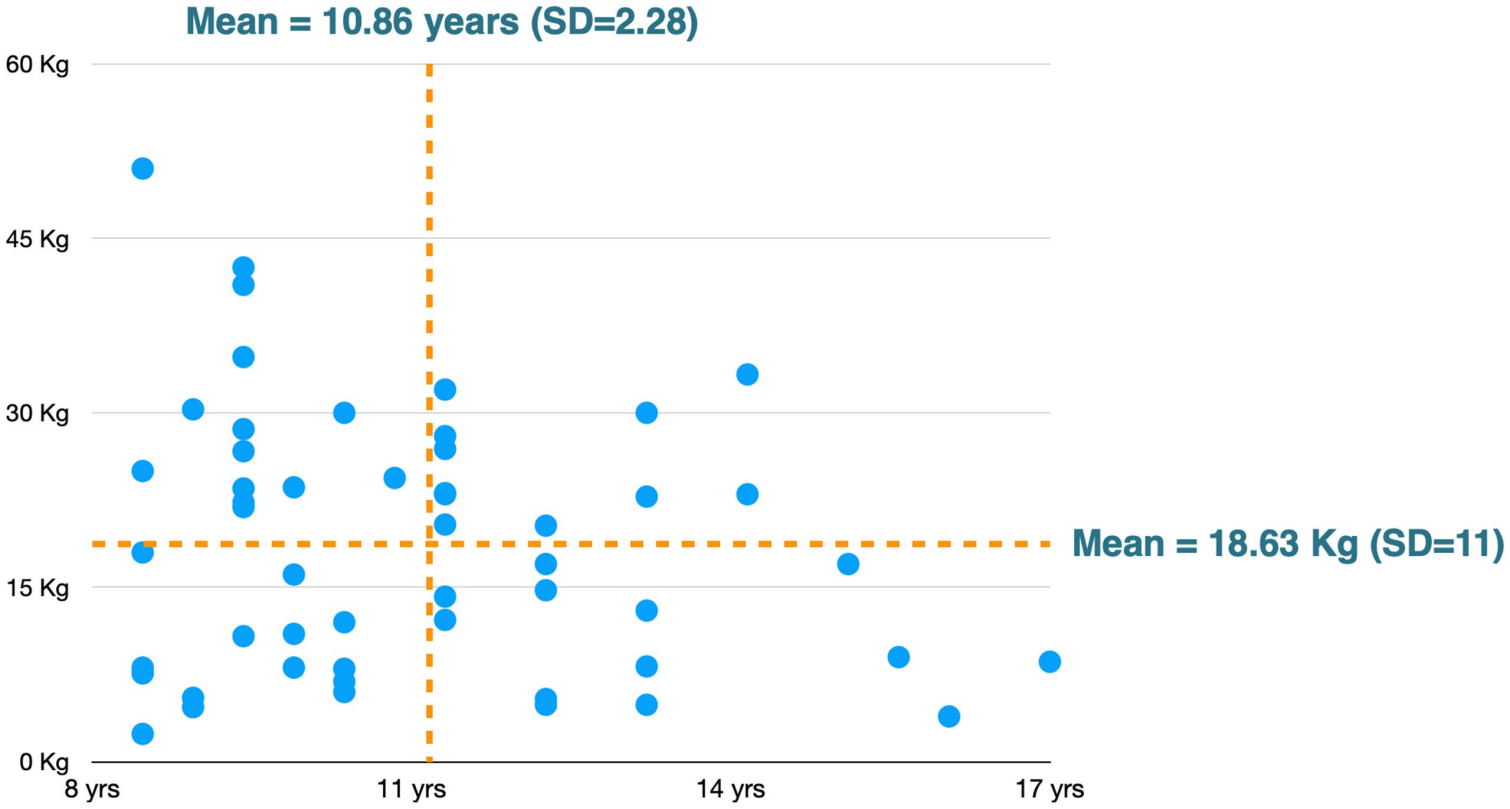
Age and weight distribution of the population.

A total of 60 dogs were recruited from three different locations (three veterinary clinics in Spain) situated in Sant Cugat, Torredembarra, and Chiva, each separated by at least 100 km. Ultimately, complete data were obtained for 57 dogs, which were included in the study. All dogs were 8 years of age or older, with a mean age of 10.86 years ± 2.28 and a mean weight of 18.63 kg ± 11.00 The age and weight distributions are shown in Figure 1. The sample was composed of 28 males and 29 females. Regarding breeds, 48% were mixed-breed and 52% were purebred, including the following: 2 Border Collies, 3 Schnauzers, 2 Golden Retrievers, 2 Yorkshire Terriers, 1 Spanish Greyhound, 1 Maltese, 1 Dalmatian, 1 German Shepherd dog, 5 Labrador Retrievers, 2 Valencian Terrier, 2 Beagles, 1 Chihuahua, 1 Fox Terrier, 2 Poodles, 1 Setter, 1 Pug, 1 German Pinscher, and 1 Spanish Water Dog.

### 3.2 Global functioning checklist

The mean GFC score was 81.0 (SD = 12.23). See Table 1 for descriptive statistics, including mean, median and standard deviation.

**Table 1:** Mean, median, minimum and maximum values for CCDR score, VSMT score, health problems score and GFC score.

	CCDR score	VSMT (mean) score	Health problems score	GFC score
Mean	36.5	2	5.7	81
StDev	39.	0.7	4.6	12.3
Median	35	2.2	5	83
Min	33	0	0	38
Max	53	3	17	98

### 3.3 Canine cognitive dysfunction rating scale (CCDR)

Based on the criteria set by the CCDR, 50 dogs exhibited no signs of cognitive dysfunction, 6 dogs scored 40 or above and were classified as being “at risk for developing CCD” (having some signs of mild cognitive dysfunction), and 1 dog scored above 50 and was classified as having severe cognitive dysfunction. Those seven dogs were designated as “affected,” and those with lower scores were designated as “unaffected” for the statistical comparisons. The overall percentage of dogs affected by cognitive dysfunction was 14%. The mean CCDR score across all dogs was 36.5 (SD = 3.90).

### 3.4 Visuo-spatial memory test (VSMT)

The mean score was 2.04 (SD = 0.73). For dogs with a CCDR score of 40 or more, the mean score was 1.46 (SD = 0.92), while the score for the single dog with a CCDR greater than 50 was 0.6. Although all fifty-seven dogs were able to complete all five trials of the VSMT, three completely failed the VSMT task, scoring zero for every one of the five trials. One of these dogs was from the affected group and two from the unaffected group.

### 3.5 Health problems score (HPS)

The mean score for the whole population was 5.67 (SD = 4.62). For dogs with a CCDR score of 40 or more, the mean score was 8.571 (SD = 4.72), and for dogs with a CCDR score below 40 it was 5.26 (SD = 4.5).

Information about the mean, standard deviation, median, minimum and maximum values for CCDR, VSMT and HPS are included in Table 1.

### 3.6 Multivariate modelling

A table of the model parameters for the five OPLS/OPLS-DA models is presented in Table 2. This includes models for VSMT Score, CCDR Score, CCDR Class, Health Problems Score and Age. For all models, two rounds of item exclusion were performed, with the presented model being the third. The number of items remaining was similar between models (8–11 items) and they were all of good quality. For the CCDR both regression and discriminant models are included. For each model, values for R^2^Y and Q^2^Y were good, and all the models were strongly significant (Table 2). The strongest model was for VSMT Score, with 57.9% of variance in VSMT Score being explained by the combination of 10 GFC items. This was closely followed by the model for age, with 57.4% of variance explained but a more robust value for Q^2^Y (0.488). The weakest model was the OPLS regression model for CCDR Score, with 41.3% of variance being explained by the combination of 11 GFC items. The CCDR score for each dog is calculated specifically to help detect whether a dog has CCD. So we decided to create a discriminant model as well as a regression model for this variable, as it seemed appropriate to do this for what had been designed to be a discriminant scale. The discriminant (OPLS-DA) model using CCDR classification (affected/unaffected) as a dichotomous variable was marginally better, explaining 50% of the variance with a combination of 10 GFC items.

**Table 2:** Model parameters for OPLS/OPLS_DA models.

Y variable	Type of model	Number of rounds of item exclusion	Number of GFC items remaining in the model	R^2^Y	Q^2^Y	*p*
VSMT score	Regression	2	10	0.579	0.425	3.2×10^−7^
CCDR score	Regression	2	11	0.413	0.35	8.8×10^−6^
CCDR class (affected vs. unaffected)	Discriminant	2	10	0.5	0.416	4.83 × 10^−7^
Health problems score	Regression	2	8	0.528	0.478	2.38 × 10^−8^
Age	Regression	2	8	0.574	0.488	7.13 × 10^−7^

The GFC items that contributed to the final OPLS/OPLS-DA models for each of the Y variables are summarized in Table 3. The loadings of each item for each model are also presented, indicating their respective contributions to the models.

**Table 3:** GFC items and loading contributing to explain the variance of VSMT, CCDR and Health Score as obtained with the different models tested.

	Model				
Item	VSMT	CCDR (Regression)	CCDR* (Discriminant)	Health problems score	Age
My dog always knows where to look to find his toys	0.375				
My dog will get off furniture when I tell him/her to	0.325	−0.259	−0.265		
It is easy to train my dog to do new things	0.295	−0.225	−0.240		
My dog is flexible and can adapt to different situations	0.250				
My dog is a good guard dog		−0.234			
My dog always recognises places that he has been before			−0.206		
My dog has good eyesight	0.199	−0.314	−0.399		−0.369
My dog has good hearing		−0.377	−0.433	−0.339	−0.447
My dog can easily find me when we are in an open space		−0.156	−0.223		
It is very easy to motivate my dog (for example, with food, play or praise)	0.466				
My dog likes to play games with me and other people he knows	0.389				
My dog is always in a good mood	0.366				
My dog stays calm and relaxed in busy environments away from home (e.g., a station or public market)	0.171				
My dog stays calm and under control when cats or wildlife are nearby on a walk			0.223		
My dog is easygoing with children					−0.281
My dog can easily jump into a car	0.254	−0.391	−0.399	−0.407	−0.345
My dog can easily climb a flight of stairs		−0.406	−0.406	−0.420	−0.359
My dog can easily walk a kilometre without tiring		−0.334	−0.344	−0.379	−0.338
My dog can easily walk five kilometres without tiring		−0.311			
My dog can easily cope with hot weather		−0.266		−0.270	
My dog can run without getting tired easily				−0.296	
My dog always gets up comfortably after sleep				−0.351	−0.338
My dog is fit and healthy				−0.367	−0.332

*Loadings are in the direction of “affected”.

### 3.7 Correlations between scores

Significant correlations were observed among several of the measures analyzed. The CCDR Score demonstrated moderate negative correlations with VSMT (r = −0.347, *p* = 0.008) and GFC_VSMT (r = −0.368, *p* = 0.005), as well as stronger negative correlations with GFC_CCDR (Regression) (r = −0.628), GFC_CCDR (Discriminant) (r = −0.504), and the GFC_Health Problems Score (r = −0.471), all of which were highly significant (*p* < 0.0002). The Health Problem Score was positively correlated with VSMT (r = 0.310, *p* = 0.019), and negatively with GFC_VSMT (r = −0.277, *p* = 0.037) and GFC_CCDR (Regression) (r = −0.535, *p* < 0.0001). The three GFC-derived measures (GFC_VSMT, GFC_CCDR, and GFC_Health Problems Score) showed strong and statistically significant intercorrelations, with coefficients ranging from r = 0.595 to r = 0.868 (*p* < 0.0001). After applying Bonferroni correction (adjusted *p*-value threshold = 0.0024), only the most robust correlations remained statistically significant. Table 4 presents the full set of correlations, with *p* values, color-coded to indicate the strength of positive or negative correlation.

**Table 4:** Correlations between each of the scores.

	VSMT		Health Problem Score		GFC_VSMT		GFC_CCDR (Regression)		GFC_CCDR (Discriminant)		GFC Health Problems Score		Age (years)	
	r	p	r	p	r	p	r	p	r	p	r	p	r	p
CCDR score	−0.347	0.008	0.310	0.019	−0.368	0.005	−0.628	<0.0001	−0.504	<0.0001	−0.471	0.0002	0.298	0.027
VSMT			−0.158	0.242	0.658	<0.0001	0.252	0.580	0.283	0.330	0.144	0.285	−0.316	0.019
Health problem score					−0.277	0.037	−0.535	<0.0001	−0.478	0.0002	−0,649	<0.0001	0.400	0.002
GFC_VSMT							0.595	<0.0001	0.659	<0.0001	0.469	0.0002	−0.460	<0.0001
GFC_CCDR (regression)									0.868	<0.0001	0.837	<0.0001	−0.392	0.003
GFC_CCDR (discriminant)											0.710	<0.0001	−0.405	0.002
GFC_Health problems score													−0.404	0.002

Bonferroni corrected *p* = 0.0018. R values are color-coded to indicate the strength of positive (green) or negative (red) correlation.

### 3.8 Comparisons between affected and unaffected dogs

**Figure 2:**
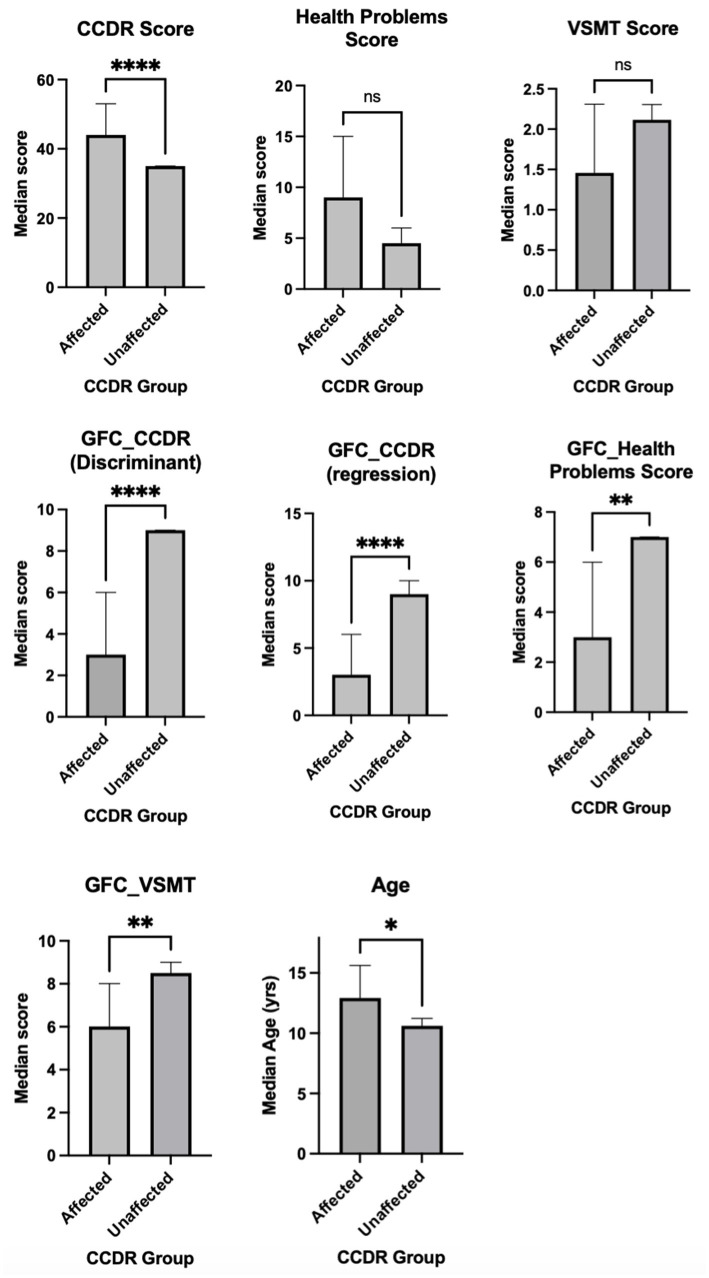
Plots for affected and unaffected scores for each significant test. For each graph, the median and its 95% confidence interval are presented (**** *p* < 0.0001, ** *p* < 0.01, * *p* < 0.05, ns not significant). Note that for the VSMT and GFC models, higher values (taller bars) indicate better performance, but for CCDR and Health Problems Scores higher values indicate being worse.

When the comparison between affected (CCDR score 40 or more) and unaffected dogs (CCDR Score below 40) was conducted for all main test scores and GFC model outputs using the two-tailed Mann–Whitney U test, statistically significant differences were found between groups for the CCDR Score (U = 0, *p* < 0.0001), with affected dogs presenting higher scores (median = 44, range = 40–53) compared to unaffected dogs (median = 35, range = 33–39). Significant group differences were also observed for the GFC-derived measures, including GFC_VSMT (U = 64.5, *p* = 0.0039), GFC_CCDR (Regression) (U = 22, *p* < 0.0001), GFC_CCDR (Discriminant) (U = 14.5, *p* < 0.0001), and GFC Health Problems Score (U = 58.5, *p* = 0.0028), all indicating worse outcomes in the affected group. No significant differences were found for VSMT (*p* = 0.051) and the Health Problem Score (*p* = 0.072), with *p*-values above the Bonferroni-corrected threshold (*p* = 0.007). There was a small but significant difference in age between the groups, with affected group being older (*p* = 0.029). This comprehensive approach to inter-group comparisons, mirroring the inter-score correlation analysis, was selected to minimize type I errors through stringent correction for multiple comparisons. The results are presented in Table 5 and Figure 2.

**Table 5:** Comparison between affected and unaffected dogs for each test.

Mann–Whitney two-tailed	Mann–Whitney U	*p*	Median affected (min-max)	Median unaffected (min-max)
CCDR score	0	<0.0001	44 (40–53)	35 (33–39)
VSMT	95.5	0.0508	1.6 (0–2.6)	2.2 (0–3)
Health problem score	101.5	0.072	9 (2–15)	4.5 (0–17)
GFC_VSMT	64.5	0.0039	6 (3–8)	8.5 (3–10)
GFC_CCDR (regression)	22	<0.0001	3 (0–6)	9 (3–11)
GFC_CCDR (discriminant)	14.5	<0.0001	3 (1–6)	9 (4–10)
GFC_Health Problems score	58.5	0.0028	3 (0–6)	7 (0–8)
Age	67.5	0.029	13 (9.5–17)	10 (8–16)

Above significant results highlighted in green, with Bonferroni corrected *p* value for outcome variables *p* = 0.007.

## 4 Discussion

This study explored whether it is possible to identify patterns of everyday behaviors that are associated with scores in validated scales and behavioral tests, using an owner-report checklist as a source of information for multivariate analysis. As a demonstration, we chose age-related changes in cognitive function, as measured with the CCDR, and visuo-spatial memory, as measured with the VSMT, as well as a measure of health, to be the measures with which to compare everyday behavior.

OPLS produced good models for each of the tests and for age. The models differed but appeared to be meaningful. In particular, the models differed from the model for age, indicating that OSC was able to deal with age as a potential confound.

The subject of aging and cognition was chosen for the study because aging in dogs, as in humans, can be conceptualized as a phenotype arising from dynamic interactions across biological and behavioral domains (Szabó et al., 2016). It appears to be a multifactorial process influenced by genetic, physiological, environmental, and experiential factors, and early signs often present as subtle changes in sensory, cognitive, or motor function (Overall, 2013). So, we were interested to see whether the method we proposed could provide contextual information about what everyday behaviors might be associated with performance in a memory test and scale for detecting canine cognitive dysfunction in an aged group of dogs. For example, to what degree are mobility and motivation, as witnessed in the everyday owner-observed patterns of behavior, associated with performance in the CCDR or VSMT.

We created multivariate models using GFC data with two validated tools, the canine cognitive dysfunction rating scale (CCDR) and the Visuo-spatial Memory Test (VSMT), and a general health score derived from a veterinary physical examination. We also produced a model relating to age, using the same method. The CCDR is designed to detect cognitive dysfunction, while the VSMT evaluates short-term visuo-spatial memory via a reward-based method (Piotti et al., 2022).

Although the GFC is an instrument that was developed for the present study, its content was informed by clinical experience and it was developed through iterative expert review. It functions as a comprehensive behavioral checklist that reflects a dog’s functional capacity. The GFC focusses on day-to-day behaviors that owners can easily report, analogous to human assessment tools like the Index of Activities of Daily Living (Katz et al., 1963). The GFC is not intended as a diagnostic tool, but rather as an exploratory measure. In this study we used the GFC as a substrate for multivariate statistics, to enable us to identify behavior patterns associated with other measures and tests. Being a very broad checklist, it does contain some items that might not apply to all dogs, or that have always been beyond a particular dog’s capabilities. For example, some owners might never have taken their dog by car and some dogs have either never been good guard dogs or live in environments where being a guard dog is never observed. However, when looking at group level data, with OPLS as a multivariate method, this is unlikely to be an issue as we are looking for overall patterns.

For our population, we recruited only dogs that were over 8 years old. Although some studies suggest cognitive changes may begin as early as 6–7 years (Studzinski et al., 2006), aging trajectories vary by breed and body size. For instance, large and giant breeds (≥22.7 kg) typically enter the senior stage between 6 and 8 years, and are considered geriatric from 9 years onward, whereas smaller breeds transition later (Bellows et al., 2015). Studies of cognition in older dogs tend to exclude all individuals with health issues. This is a good way to minimize the confounding effects of ill-health, but it does create a population that is unusual. In this study, we chose to include a wide range of dogs of different types, excluding only dogs with significant health issues. This meant that the population was more typical of a group of owned dogs of this age, with the dogs having minor health issues.

In our sample, 14% of dogs met the CCDR threshold for some degree of cognitive dysfunction, which aligns with previous prevalence estimates (12%) (Salvin et al., 2010). Given the modest prevalence of this condition, we would have needed a much larger sample size to get a large group of affected dogs, and other authors have reported difficulty in recruiting dogs with more advanced impairments (Watowich et al., 2020).

When performing studies with pet dogs, it is challenging to obtain samples that are representative of a wide range of breeds and sizes. Nevertheless, such populations still tend to be more diverse than those used in laboratory dog studies. The population in the present study included an almost equal proportion of male and female dogs, a mix of pure-bred and mixed-breed dogs, and a range of bodyweights. So, although small, our population is reasonably representative of an aged dog population.

Multivariate modelling was effective and consistent across the test models, with the same number of rounds of exclusion and a very similar number of items remaining after feature selection. All models were moderately strong, with the strongest being for VSMT (R^2^Y = 0.579). This was surprising, given that the VSMT is a laboratory test of memory that might not be expected to have obvious correlations with everyday behaviors. The weakest model was the regression model for CCDR score, with a modest R^2^Y of 0.413. Perhaps the difference in quality between this and the discriminant CCDR model is due to the CCDR having been mathematically optimized to discriminate between groups for case detection, with different loadings for certain items.

To aid in interpretation of the differences between the models, we informally categorized items in the subscales into four domains: cognition and memory, sensory function, motivation and mood, and physical health (see Table 6). Distinct patterns emerged: motivation-related items were more strongly associated with VSMT scores, while sensory and physical health items predominated in CCDR models. Only one item—"My dog can easily jump into a car”—was present in all models, possibly indicating its value as a composite indicator of mobility, engagement, and perhaps cognitive capacity.

**Table 6:** GFC item classification according to their primary component.

	Model				
Item	VSMT	CCDR (regression)	CCDR* (discriminant)	Health problems score	Age
My dog always knows where to look to find his toys	0.375				
My dog will get off furniture when I tell him/her to	0.325	−0.259	−0.265		
It is easy to train my dog to do new things	0.295	−0.225	−0.240		
My dog is flexible and can adapt to different situations	0.250				
My dog is a good guard dog		−0.234			
My dog always recognises places that he has been before			−0.206		
My dog has good eyesight	0.199	−0.314	−0.399		−0.369
My dog has good hearing		−0.377	−0.433	−0.339	−0.447
My dog can easily find me when we are in an open space		−0.156	−0.223		
It is very easy to motivate my dog (for example, with food, play or praise)	0.466				
My dog likes to play games with me and other people he knows	0.389				
My dog is always in a good mood	0.366				
My dog stays calm and relaxed in busy environments away from home (e.g., a station or public market)	0.171				
My dog stays calm and under control when cats or wildlife are nearby on a walk			0.223		
My dog is easygoing with children					−0.281
My dog can easily jump into a car	0.254	−0.391	−0.399	−0.407	−0.345
My dog can easily climb a flight of stairs		−0.406	−0.406	−0.420	−0.359
My dog can easily walk a kilometre without tiring		−0.334	−0.344	−0.379	−0.338
My dog can easily walk five kilometres without tiring		−0.311			
My dog can easily cope with hot weather		−0.266		−0.270	
My dog can run without getting tired easily				−0.296	
My dog always gets up comfortably after sleep				−0.351	−0.338
My dog is fit and healthy				−0.367	−0.332

* Loadings are in the direction of “affected”.

**Table:** 

Cognition & memory	
Sensory function	
Motivation & mood	
Physical health	

It appears that multivariate regression modelling using PLS with an integral orthogonal signal correction (OSC) filter was able to produce models that were specific to CCDR score, VSMT score, health score and age. The age model was quite different pattern from both the CCDR and VSMT models, indicating that the OSC filter was able to reject the effects of age when modelling those variables. It is interesting that the Health Problems Score model was most similar to the age model.

Table 7 shows a breakdown of the different categories of items that contributed to each model. This illustrates the differing balance of items between the models. The VSMT model includes mostly items relating to cognition & memory and motivation & mood, but the Health Problems Score model almost exclusively included physical health items that was most similar to the age model. The CCDR models were a mix of items.

**Table 7:** A breakdown of the types of GFC items that contribute to the models.

	Model				
	VSMT	CCDR (regression)	CCDR (discriminant)	Health problems score	Age
Cognition & memory	4	3	3	0	0
Sensory function	1	3	3	1	2
Motivation & mood	4	0	1	0	1
Physical health	1	5	3	7	5

Eight out of ten of the GFC items that contributed to the VSMT model were related to cognition & memory or motivation & mood. Our methodology revealed that, in older dogs, cognition or memory related everyday abilities, such as knowing where to find toys, obeying certain commands, being easy to train and adaptable to situations, were positively associated with better VSMT performance. Being easily motivated by reinforcers such as food or praise, being playful, and having a consistently positive mood were also associated with better VSMT performance. This makes sense and would be what we might expect for a dog of any age. The single sensory function item in the VSMT model was “my dog has good eyesight,” which is understandable given that this is a visuo-spatial task and the dogs are old enough for some individuals to have a degree of visual impairment. Good vision and the ability to jump into a car are, perhaps, more age-related items that could be less influential in younger dogs. This gives us an indication of the kind of older dog that is likely to perform well, or badly, in the VSMT and it makes sense that an older dog that has good mobility, is easily motivated, quick to learn and has good vision would perform well in a visuo-spatial memory test.

Modelling VSMT scores with the GFC has provided useful insights into the attributes that contribute to performance in this test in older dogs. The multivariate model was strong enough that when a score was calculated for the resultant GFC_VSMT subscale, that score correlated strongly with VSMT scores (r = 0.658).

In the Health Problems Score model, all loadings of GFC items were negative, indicating that performance for these items declined with an increased number of health problems. Seven out of eight of the GFC items that contributed to the Health Problems Score model were those which reflected reduced mobility (implying absence of pain) and reduced exercise tolerance, with the strongest negative loadings being for “My dog can easily climb a flight of stairs,” “My dog can easily jump into a car,” and “My dog can easily walk a kilometer without tiring.” These items primarily reflect mobility and endurance, but they could also indirectly capture the impact of chronic pain on emotional and behavioral regulation (Mills et al., 2020b; Fatjó and Bowen, 2020b). There was also a negative loading for “My dog has good hearing,” which is interesting because an association has been found between hearing impairment and reduced walking endurance in people (Martinez-Amezcua et al., 2021), and people with hearing loss have been found to become physically inactive faster than those without (Goodwin et al., 2023). In addition, there is evidence that the same processes of cartilage degeneration that affects mobility and causes pain in osteoarthritis can affect the joints within the middle ear and lead to hearing loss (Rawool and Harrington, 2007). These are speculations, but there does appear to be a complex link between endurance, arthritis and hearing loss in people that has not been investigated in dogs.

The age model was quite similar to the Health Problems Score model, with most items being in the physical health category. Unlike the health problems score mode, both hearing and eyesight were negatively associated with age. So, the picture is that increased age is associated with a loss of stamina, mobility and strength, as well as sensory ability. Age was also the only model to include an association with the GFC item “My dog is easygoing with children,” albeit negative. Clients to sometimes mention that their older dogs have become less tolerant of children, and it is interesting that this is related to some specific characteristic of age, that is different from what is measured by CCDR, VSMT or a health measure.

Two models were created using the CCDR, one regression and one discriminant comparing dogs that were classified as affected (score 40+) or unaffected (score 50+) based on CCDR score.

None of the items in the CCDR itself directly relate to aspects of physical health or motivation, they relate to abnormal repetitive behavior, confusion, disorientation, and dysfunctions of sensory and cognitive systems. Items relating directly to memory are also notably absent from the CCDR. It could be argued that failure to recognize familiar people or pets relates to memory, but it is equally an indication of sensory or cognitive impairments.

The scoring scheme for CCDR also includes a 2x multiplier when scoring the item “Compared with 6 months ago, does your dog have difficulty finding food dropped on the floor” and a 3x multiplier when scoring the item “Compared with 6 months ago, does your dog fail to recognize familiar people or pets.” This boosts the influence of poor visual and olfactory perceptual performance within the scale, which further shifts the scale toward measuring cognitive and perceptual systems rather than memory. This may be why there was not a significant correlation between CCDR and VSMT scores and there was not a significant difference between the VSMT scores in affected and unaffected dogs.

In the CCDR regression model, all loadings of GFC items were negative, indicating that performance for these items reduced with an increase in CCDR score. Five out of eleven GFC items in this model were connected with physical health, including “My dog can easily climb a flight of stairs” and “My dog can easily walk five kilometers without tiring.” This may be because physical aging tends to be more apparent in dogs with mental aging, or vice versa. However, it could also indicate that physical health problems, such as pain, impaired mobility and exercise intolerance, are responsible for some of the owner-observed changes measured by the CCDR. It would be interesting to study whether improvements in pain and mobility with analgesic and anti-inflammatory drug treatment are associated with improvements in CCDR score for dogs that are not receiving specific treatments for CCD.

Both the regression and discriminant CCDR models shared only four items with the VSMT Scores model, and neither included any of the motivation & mood items from the VSMT Scores model. It may be that the three cognition & memory GFC items that negatively load in the CCDR Regression model are those which are more susceptible to aging, compared with the other two GFC items that are not shared with the VSMT model. However, “My dog has good hearing” had a strong negative loading in the model, and a loss of hearing could be associated with poor performance on the GFC cognition & memory items “My dog will get off furniture when I tell him/her to,” and “it is easy to train my dog to do new things.” It is interesting that “My dog is a good guard dog” has a negative loading in the model, as that could relate to failure of a dog to be able to distinguish between familiar and unfamiliar people, which would be in line with that aspect of the CCDR. Both “My dog has good hearing” and “My dog has good eyesight” have negative loadings in the model, and this may be because owners interpret failure to recognize familial people, pets and places, as well as bumping into doors or getting stuck in places as signs of sensory impairment.

It should be noted that the items in CCDR mostly relate to quite severe pathological behavioral changes that reflect gross disturbances of sensory, perceptual and cognitive systems. On the other hand, the items in GFC relate to a wide range of everyday capabilities. So, one would not expect to find a set of GFC items that are even closely equivalent to CCDR. However, it is striking that CCDR can be modelled so well using GFC, adding context to the meaning of the CCDR and what may be contributing factors to poor scores.

It is interesting that three of the physical health items of the GFC contributed to all of the models apart from VSMT; My dog can easily jump into a car,” “My dog can easily climb a flight of stairs,” and “My dog can easily walk a kilometer without tiring.” These are three simple questions that could be asked of any owner of an older dog, either to screen for age-related change in general or as an complement to a scale like the CCDR, in order to provide additional information about the contribution of physical health to the presentation of suspected case of CCD. These three items also resonate with the “instrumental activity for daily quality of life” checklist (IADQOL) in the paper on canine geriatric rehabilitation and functional scoring by Frye et al. (2022). This includes three specific measures of mobility: “ascending/descending a full flight of stairs,” “Moving in and out of a vehicle,” and “walking short distances outside.” It also included items relating to play, the ability to navigate to a play of rest and the ability to maintain control and urination and defecation for 6-8 h.

Each model resulted in a subscale of GFC items that could then be scored so that scores could be compared between CCD affected and unaffected dogs, and correlations could be tested between the various scores and test results.

Only VSMT and Health Problem Score did not differ significantly between CCDR affected and unaffected groups. There was also no significant correlation between Health Problem Score and CCDR score. The lack of difference in Health Problem Score between groups is good, as it indicates that overall health was not a confounding factor between affected and unaffected groups, and the lack of correlations between Health Problem Score and CCDR supports a lack of association between CCDR and general health.

The lack of difference in VSMT is a surprise, given that CCDR has been validated for the detection of CCD. The VSMT has been used to evaluate brain aging and one would expect the CCDR to be sensitive to memory impairment. This surprising result may be due to the multifactorial nature of aging, and that different measures evaluate different aspects of brain aging. The only items in the CCDR that might relate to memory are those about failure to recognize familiar people or pets. So, perhaps CCDR when discriminating between affected and unaffected individuals, it is relying on cognitive processes that relate to processing and recognition rather than the short-term working memory measured by VSMT. In addition, the CCDR is a relatively conservative tool in its classification: dogs must exhibit substantial impairment across several domains to receive a “positive” score. This threshold may result in reduced sensitivity to early or domain-specific changes, such as subtle memory decline detectable by the VSMT, particularly in dogs who do not yet meet the CCDR cutoff but may still be experiencing functional changes.

Values for all of the GFC subscales differed significantly between unaffected and affected dogs, but with higher values of U than for CCDR score. This is to be expected, given that the groups were identified using the CCDR cutoff point.

There were significant moderate to strong correlations between all the GFC subscale scores, which is to be expected when there was some degree of overlap between the items in the subscales.

Although the original purpose of the study was to test a methodology, we expected that the resulting subscales would themselves have some practical value, both scientifically and clinically. Scientifically, in older dogs the GFC_VSMT subscale could be used to screen and select suitable participants for studies using the VSMT (or similar memory tests), or to match groups in an intervention study to reduce confounding factors. With memory/cognitive studies involving owned dogs, it could be used as a secondary outcome measure to evaluate everyday changes in behavior in response to dietary and drug interventions. Clinically, it could be used to detect dogs with behavioral characteristics consistent with the onset of memory-related cognitive decline. This could be as a precursor or supplement to using the CCDR, as it includes items that are distinct from those in the GFC_CCDR subscale.

When comparing scores between affected and unaffected dogs, it was not ideal to make such a comparison between two groups of such difference in size. However, we felt that it should be done for completeness, because the purpose of the CCDR was to discriminate between affected and unaffected dogs. However, this weakness has to be considered when interpreting the results.

Comparing CCDR and VSMT scores, no significant differences in VSMT performance were observed between CCDR-affected and unaffected dogs after Bonferroni correction. One possible explanation is that the VSMT relies heavily on food motivation; performance may be more closely tied to individual or group differences in engagement and drive rather than cognitive impairment. Additionally, the task’s low physical demands may minimize the impact of physical limitations on performance. So, a highly motivated dog with bad health status probably can perform better in the VSMT than the CCDR, even with the same cognitive impairment.

The domain-based classification reinforced this differentiation: VSMT-related subscales emphasized memory and motivation, whereas CCDR subscales encompassed cognitive, sensory, and physical domains. The limited overlap between models suggests these tools capture distinct facets of the aging process. The CCDR, in particular, may offer a broader assessment encompassing both functional and sensory decline.

It is interesting that from both the regression and discriminant models, the resulting GFC_CCDR subscales included more items relating to sensory and physical function than the GFC_VSMT did. Both forms of the GFC_CCDR included hearing as well as vision, being easily able to walk one kilometer and climb a flight of stairs in addition to being easily able to jump into a car. The GFC_CCDR subscale from the regression model also included being easily able to walk 5 kilometers and being able to cope with hot weather. The anti-correlation between score for this subscale and CCDR score was strong (r = −0.628). The implication is that a substantial proportion of the variance in CCDR scores can be explained by an individual’s vitality (i.e., activity, mobility and stamina). Again, it seems that the two forms of GFC_CCDR, particularly the one produced by the regression model, could have scientific and clinical applications. For example, if CCDR is used as primary outcome measure in a biological intervention study for brain aging, GFC_CCDR (Regression) could be used to exclude dogs with vitality issues that would not respond to the treatment and could confound the results. In a clinical setting, administering GFC_CCDR (Regression) alongside CCDR would help the clinician to detect underlying vitality and sensory issues that could contribute to a falsely elevated CCDR score.

Similarly, an 11-item GFC subscale correlated with VSMT scores. Dogs with higher VSMT scores also tended to have higher GFC scores, suggesting a behavioral link with cognitive performance and better day-to-day performing. The perception of the dog’s intelligence is mediated by the relationship with them (Howell et al., 2013). It may be possible to infer a dog’s cognitive status by using carefully designed questions targeting relevant everyday behaviors, thereby reducing the need for cognitive tests that are often impractical to administer in home environments.

Despite these limitations, the methodological framework presented here offers a scalable, low-cost approach for identifying functional decline in aging dogs. With further research, the GFC could complement clinical assessments and contribute to more proactive, holistic care strategies in geriatric veterinary medicine. Future studies should also explore associations between positively scored behaviors and measures of psychological well-being or quality of life in aging dogs. Longitudinal designs could also assess the impact of preventive care or environmental enrichment on functional aging.

### 4.1 Limitations and further work

The sample size of this study was quite small, as was the proportion of dogs meeting the threshold for cognitive dysfunction. Potential selection bias also exists, as participating owners may have been more observant or engaged than the general population. Furthermore, no demographic or lifestyle data were collected, such as diet, exercise, or cognitive stimulation, all of which may affect behavioral aging. However, this background data is also missing from other studies of aging in dogs. Future studies could include a more complete health screen, including urinalysis.

Although the methodology we used produced interesting results that indicate that it could be used to add context to any study of aging that uses behavioral measures or scales, further studies would be needed to investigate whether the subscales we found in our study could be used as independent tools to detect CCD or to screen populations for studies.

The methodology we used is not limited to older dogs and behavior tests or scales, it could also be with biological parameters such as thyroid function, basal cortisol or neuter status, and it could be used to profile behaviors in younger dogs.

## 5 Conclusion

This study did not aim to validate a new behavioral instrument, but rather to investigate whether statistical modelling techniques could help relate owner-reported behaviors to validated clinical assessments. The findings suggest that the approach worked, and that such modelling could be used to extract meaningful subscales from broader observational data. With further development and larger samples, this approach may contribute to the early detection of age-related changes, and support research into healthy aging in companion dogs.

## Data Availability

The raw data supporting the conclusions of this article will be made available by the authors, without undue reservation.
