# Supplementary material for: Which aspects of the everyday behavior of older dogs correlate with performance on a visuo-spatial memory test and the canine cognitive dysfunction rating scale (CCDR)?
Source: Front Aging Neurosci. 2026 Feb 3;18:1678032. doi: 10.3389/fnagi.2026.1678032 (PMC12910368; doi:10.3389/fnagi.2026.1678032)
Supplement: Supplementary file 1 [file Data_Sheet_1.pdf]

Movement: Limp
Movement: Stiffness
Movement: Paresis
Fur: Altered
Fur: Poor condition
Fur: Alopecia
Skin: Altered
Skin: Superficial inflammation
Skin: Wounds
Skin: Peeling
Skin: Pruritus
Skin: Hyperpigmentation
Skin: Nodules
Abdomen: Distended
Eyes: Altered
Eyes: Squint
Eyes: Loss of corneal transparency
Eyes: Epiphora
Eyes: Conjunctivitis
Cough
Vomiting
Diarrhoea
Sneezing
Incontinence
Lymph nodes: Altered
Lymph nodes: Local lymphadenomegaly
Lymph nodes: General lymphadenomegaly
Presence of tumors
Muscular: Tone altered
Muscular: Local atrophy
Muscular: General atrophy
Physical exam: Pain present
Abdomen: Rigid
Abdomen: Distended
Pulse: Altered
Pulse: Not palpable

Pulse: Weak
Proprioception: Altered
Testicles: Altered
Testicles: Different sizes
Mammaries: Altered
Mammaries: Nodules
Respiratory auscultation: Altered sounds
Cardiac Auscultation: Altered sounds
Cardiac Auscultation: Murmur
Cardiac Auscultation: Bradycardia
Cardiac Auscultation: Tachycardia
Ears: Pinna altered
Ears: Pinna dirty
Ears: Pinna inflammation
Ears: Pinna bad odor
Ears: Pinna pain
Ears: Outer ear altered
Ears: Outer ear dirty
Ears: Outer ear irritation
Ears: Outer ear pain
Pads: Altered
Nails: altered

The list of items included in the health score. Note that the examination covered all body systems in detail, and this is the set of items for which at least one dog scored positively. “Altered” referred to a feature that departed from normality in some way that had not been covered by specific items in the checklist.
