# Supplementary material for: Which aspects of the everyday behavior of older dogs correlate with performance on a visuo-spatial memory test and the canine cognitive dysfunction rating scale (CCDR)?
Source: Front Aging Neurosci. 2026 Feb 3;18:1678032. doi: 10.3389/fnagi.2026.1678032 (PMC12910368; doi:10.3389/fnagi.2026.1678032)
Supplement: Supplementary file 2 [file Data_Sheet_2.pdf]

| |
|---|
| My dog only goes to the toilet in the right places, never in the house |
| I can easily take food away from my dog |
| My dog will drop food or things that he/she has picked up in the street when I tell him/her |
| My dog only chews and plays with his/her own toys, never my possessions |
| My dog will drop things he has picked up in the house when I tell him/her |
| My dog obeys commands, like recall or sit, no matter where we are and what is happening |
| My dog walks calmly next to me on the leash, without pulling |
| My dog is calm and relaxed when travelling in a vehicle |
| My dog will get off furniture when I tell him/her to |
| My dog can easily be left alone at home without causing any trouble |
| My dog can easily be left to wait outside a shop or in the car |
| My dog is easygoing with everyone he/she meets in the street or park |
| My dog is easygoing with everyone who visits our home |
| My dog settles down quickly and is relaxed when a familiar person is visiting our home |
| My dog settles down quickly and is relaxed when a stranger is visiting our home |
| My dog settles down quickly and is relaxed when we have a gathering of people at our home |
| My dog settles down quickly and is relaxed when I stop to talk with someone on a walk |
| When my dog goes to approach someone, I can easily call him/her away |
| My dog will sit if a stranger asks him/her to |
| My dog is easygoing with new dogs |
| My dog is easygoing with familiar dogs |
| My dog walks away if other dogs are difficult or aggressive |
| My dog likes to play with other dogs |
| My dog lets me groom him without any difficulty |
| My dog lets me clip his/her claws without any difficulty |
| My dog lets me pick him up without any difficulty |
| The vet finds it easy to examine my dog |
| My dog is easy to take to the vet clinic |
| My dog is calm and relaxed in the waiting room of the vet clinic |
| My dog sleeps through the night without disturbing anyone |
| My dog is not frightened by loud noises |
| My dog is not frightened by sudden noises |
| My dog is not frightened by angry or raised voices |
| My dog is not afraid of fireworks and/or thunderstorms |
| My dog stays calm and under control when cats or wildlife are nearby on a walk |
| My dog stays calm and under control when a jogger, cyclist or skateboarder goes past |

| |
|---|
| My dog stays calm during household activities like vacuum cleaning, cooking and gardening |
| My dog stays calm and relaxed in busy environments away from home (e.g. a station or public market) |
| My dog stays calm and relaxed in new and unfamiliar environments |
| My dog makes friends easily with new people |
| My dog makes friends easily with new dogs |
| My dog never tries to steal food from the table |
| My dog never tries to steal food from people whilst they are eating |
| When my dog goes to approach a dog, I can easily call him/her away |
| When my dog goes to chase something, I can easily call him/her away |
| It would be easy for someone else to take care of my dog |
| My dog copes easily with going on holiday |
| My dog is easygoing with children |
| My dog remembers people after meeting them only once |
| My dog always recognises people that he knows well |
| My dog always recognises places that he has been before |
| My dog likes to play games with me and other people he knows |
| My dog always knows where to look to find his toys |
| My dog always remembers the way back home |
| My dog can easily find me when we are in an open space |
| My dog knows the name of individual toys |
| My dog can always find food dropped on the floor |
| It is easy for me to get my dog's attention |
| My dog always waits or sits at the kerb before crossing the road |
| My dog stays calm and controlled when we are about to go for a walk |
| My dog stays calm and controlled when I am preparing his food |
| My dog always leaves dogs alone if they doesn't want to play |
| My dog isn't excited by sudden movements or sounds |
| My dog can easily climb a flight of stairs |
| My dog can easily jump into a car |
| My dog can easily walk a kilometre without tiring |
| My dog can easily walk five kilometres without tiring |
| My dog can run without getting tired easily |
| My dog can easily cope with hot weather |
| My dog has good eyesight |
| My dog has good hearing |
| My dog always gets up comfortably after sleep |

| |
|---|
| My dog isn't easily disturbed once he is asleep |
| My dog settles easily before going to sleep |
| When my dog wakes up, he is always in a good mood |
| My dog is fit and healthy |
| My dog's behaviour always makes sense |
| My dog is always willing to be petted by familiar people |
| My dog does not run away when off leash |
| I'm confident that my dog would never bite anyone |
| My dog does not bark when left alone |
| My dog does not damage things when left alone |
| My dog is a good guard dog |
| My dog always keeps his excitement in check |
| My dog stays calm regardless of the situation |
| My dog remains easy to control even when he is excited |
| My dog's personality is always the same, even when he is excited |
| My dog asks me when he really needs something, but doesn’t pester me |
| My dog finds it easy to wait |
| My dog finds it easy to resist reacting to things |
| My dog plans what he/she is going to do |
| It is very easy to motivate my dog (for example, with food, play or praise) |
| My dog is always curious about new things |
| My dog likes to be trained |
| My dog is always interested in people he knows |
| My dog is very sociable with other dogs |
| My dog is very sociable with people |
| My dog tends to see the good in situations, rather than the bad |
| My dog is always in a good mood |
| My dog only avoids situations that are genuinely risky |
| My dog is always good tempered even when he is not getting what he wants |
| My dog stays calm even when he is not getting what he wants |
| My dog recognises my moods and emotions |
| My dog tries to help me when I am unhappy |
| My dog tends to pick up good, rather than bad habits |
| It is easy to train my dog to do new things |
| My dog is flexible and can adapt to different situations |
| My dog is flexible and can adapt to changes of routine |

| |
|---|
| I always know what my dog is going to do next |
| My dog quickly learns what he is not allowed to do |
| My dog looks to me for guidance whenever he is uncertain what do |
| My dog is always easy to live with |
